# Selection of Base Materials for Repair Welding Using BWM-TOPSIS and BWM-RADAR Approaches

**DOI:** 10.3390/ma18245696

**Published:** 2025-12-18

**Authors:** Dušan Arsić, Djordje Ivković, Ranka Sudžum, Dragan Marinković, Nikola Komatina

**Affiliations:** 1 Faculty of Engineering, University of Kragujevac, 34000 Kragujevac, Serbia; dusan.arsic@fink.rs (D.A.); djordje.ivkovic@fink.rs (D.I.); nkomatina@kg.ac.rs (N.K.); 2 Faculty of Mechanical Engineering, University of East Sarajevo, 71123 East Sarajevo, Bosnia and Herzegovina; ranka.gojkovic@ues.rs.ba; 3 Department of Structural Analysis, Technical University of Berlin, 10623 Berlin, Germany; 4 University College, Korea University, 145 Anam-ro, Seongbuk-gu, Seoul 02841, Republic of Korea; 5 Institute of Mechanical Science, Vilnius Gediminas Technical University, LT-10105 Vilnius, Lithuania

**Keywords:** base material, repair welding, sustainability, MADM, BWM, TOPSIS, RADAR

## Abstract

In this paper, the selection of the optimal base material to be used in the repair welding process is presented. The aim of the study was to determine which of the available materials has the best characteristics, based on an analysis conducted in a company engaged in construction works. Three base materials were considered in the study: ABRADUR 58, E DUR 600, and CrWC 600 electrodes. Repair welding was performed on components for a construction machinery facility using the manual metal arc welding procedure. For the selection of the optimal base material, a combined Multi-Attribute Decision-Making (MADM) approach was applied. The base materials were evaluated based on four attributes: wear track width, cost, mass loss, and hardness of welded layers. The Best–Worst Method (BWM) was used to determine the attribute weights, while the Technique for Order of Preference by Similarity to Ideal Solution (TOPSIS) and Ranking based on the Distances And Range (RADAR) methods were applied in parallel for the ranking and selection of base materials. The analysis showed that in the considered case, the E DUR 600 electrode was the most suitable choice, which was confirmed through the application of both the TOPSIS and RADAR methods.

## 1. Introduction

Engineers in industrial practice frequently encounter various problems where decisions made at a given moment can directly affect product quality, the execution of production processes, and even the overall business performance of the company. One common challenge faced by engineers, particularly designers and process engineers, is material selection. From raw materials used in batch and mass production to materials for individual components and those applied in various processing operations, engineers must consistently find a balance among numerous often conflicting characteristics, such as cost, quality, machinability, availability, aesthetic appeal, and more.

Perhaps most importantly, material selection has a direct impact on the mechanical performance and functional characteristics of a product [1]. In this study, the focus is on the selection of a base material for repair welding, demonstrated through a practical case involving components for a construction machinery facility. The process under consideration is manual metal arc welding, and the primary objective is to select a base material that ensures the repaired working component can be used again without compromising its functional properties. At the same time, the goal is to enable the company to achieve financial savings, while also contributing on a global scale to a reduction in the resources and energy required for the production of new steel components and parts.

The selection of an appropriate base material in repair welding is one of the key factors determining the success of the procedure, the service life of the repaired component, and the overall economic feasibility of the intervention. The quality of a repair largely depends on the choice of a suitable base material, which must be compatible with the base and the operational conditions [2]. An improper selection of the base material can lead to various types of damage, such as microcracks, delamination, insufficient adhesion between layers, or accelerated wear. This not only negates the effect of the repair but also increases maintenance costs. Therefore, in industrial practice, it is essential to apply a systematic approach that includes both chemical and mechanical characterization of the materials, as well as a realistic assessment of wear conditions (abrasive and impact), in order to define the optimal combination of hardness and toughness for the deposited layer.

In this study, three Multi-Attribute Decision-Making (MADM) methods were applied. The Best–Worst Method (BWM) was used to determine the attribute weights. This method, defined in [3], is one of the more recent approaches within this group of methods. It is based on assessing the relative importance of attributes by comparing all attributes with the best and worst attribute. The method relies on solving a linear programming problem, following the rules defined in the conventional approach. Although relatively new, it has been widely applied in the relevant literature; however, its application in material selection problems remains limited. The main advantage of this method is that it requires decision-makers to perform pairwise comparisons only between the best and worst attribute and all others, rather than comparing every attribute with every other attribute, as is the case with some other methods such as Analytic Hierarchy Process (AHP) [4]. In this way, it is much easier for decision-makers to provide consistent evaluations, especially in problems involving a larger number of attributes.

For the ranking of base materials, two MADM methods were applied in parallel. The first was the Technique for Order of Preference by Similarity to Ideal Solution (TOPSIS) [5], and the second was the RAnking based on the Distances And Range (RADAR) method [6]. 

There are two key reasons why these two methods were selected. The first reason is that the TOPSIS method is the most widely used approach in the relevant literature for solving ranking problems, and its reliability has been proven many times. On the other hand, the RADAR method is a relatively new MADM approach that has been gradually gaining attention and application. In this case, an established and a new approach are compared for solving the same class of problems.

The second reason is that these two methods are based on different mathematical foundations. The TOPSIS method is fundamentally a compensatory approach, meaning that an alternative can compensate for a poor score in one attribute with a higher score in another. In contrast, the RADAR method, although it shares some characteristics with compensatory approaches, also exhibits non-compensatory properties and is capable of penalizing alternatives with extreme values, particularly with respect to the most important attributes.

The aim of this research is to demonstrate that the combination of BWM-TOPSIS and BWM-RADAR will yield reliable, stable, and consistent ranking results for the considered base materials with similar characteristics. Based on the previous experience and knowledge of the company’s experts, three materials were shortlisted: ABRADUR 58, E DUR 600, and CrWC 600. Since their characteristics are quite similar and difficult to compare directly, the company’s experts identified four key attributes used to evaluate the base materials: wear track width, cost, mass loss, and hardness of welded layers. More detailed explanations of the considered characteristics and base materials are provided in the following sections of this paper.

As these methods are well established in the literature, including the RADAR method, which is relatively new. It should be emphasized that the contribution of this work does not lie in advancing the existing methodology, but rather in integrating and comparing the two proposed models, which has not previously been achieved in the context of base material selection. Therefore, from a methodological perspective, the contribution lies in defining a structured framework based on the parallel application of two approaches—one compensatory (TOPSIS) and one partially compensatory (RADAR)—with mutual comparison and validation of the obtained results. Thus, this study does not introduce a new method, but proposes a robust decision-making model.

The practical contribution of this research is reflected in the application of the proposed model to solve a real-world problem in an industrial environment. At the same time, it was necessary to ensure that the model is not only sufficiently reliable but also clear and understandable to practitioners, enabling them to easily apply it in the future when addressing similar problems they encounter on a daily basis.

After the Section 1, the Section 2 provides a literature review in the field of applying MADM methods to material selection problems in industry. The Section 3 explains the applied methodology and presents the steps for implementing the selected MADM methods. The Section 4 presents the case study, while the Section 5 highlights the main conclusions of the research.

## 2. Literature Review

The selection of materials represents an important research problem in engineering. In addition to technical aspects, the selection of a base material increasingly involves considerations of sustainable development and resource efficiency. As highlighted in the study [7], the authors applied the VIKOR (in English: Multi-Criteria Optimization and Compromise Solution) and TOPSIS methods in the steel production industry, demonstrating that proper management of raw materials and material selection directly affect waste generation and the overall sustainability of production. In this context, choosing a base material that ensures a longer service life and reduces the need for frequent repairs represents not only a technical but also an economic and ecological justification. For this reason, the rational selection of a base material is not merely a matter of performance but also an integral part of a broader strategy for optimizing material consumption, reducing waste, and enhancing the reliability of machinery under operational conditions.

A significant number of studies in the relevant literature have addressed this issue, where authors have applied various MADM approaches for material evaluation and selection for different purposes. In [8], the authors provided a detailed overview of the applied approaches for material selection and classified them into six main categories: (1) screening methods, (2) choosing and ranking methods, (3) artificial intelligence, (4) optimization and mathematical methods, (5) strategies, and (6) fuzzy and uncertainties. According to the same source, the most frequently used category is choosing and ranking methods (40.51%), which is also the focus of this study.

The problem addressed in this paper, namely the selection of base materials for welding, has not been extensively explored in the relevant literature so far. To date, only one study of this kind has been conducted. In paper [9], the authors applied a hybrid MADM approach for the selection of spot welding electrode materials used in the automotive industry. In that study, the AHP method was used for grading the electrode properties, while TOPSIS and Simple Additive Weighting (SAW) were employed for material ranking.

Although the paper in which the BWM was first introduced and its foundations established was published ten years ago, this method has found application in numerous research problems, including material selection. It has primarily been used for determining the weights of attributes, often in combination with other MADM methods. For instance, BWM has been applied to determine attribute weights in material selection for product prototyping within additive manufacturing [10], for coating material selection in tooling industries [11], construction material selection [12,13], and phase change material selection [14]. 

The TOPSIS method has also been used in the relevant literature for ranking and selecting materials. Some of the application areas of TOPSIS for material selection include additive manufacturing [15], manufacturing process and tooling material selection [16], eco-materials for furniture production [17], the automotive industry [18], the energy sector [19], and the pulp and paper industry [20], among others. Interestingly, to date, the combination of BWM and the TOPSIS method has not been applied in the literature to solve this type of problem. 

As already mentioned, the RADAR method is a new approach, whose foundations were first presented about a year ago. To date, it has not been applied in the literature for material selection problems, but the authors have already used it to address other ranking problems, such as the selection and prioritization of KPIs that best reflect the efficiency and sustainability of business processes [21], evaluation of leadership styles in multinational corporations [22], and the selection of tractors in green ports [23]. In addition, the RADAR method has been recognized as one of the new approaches that has already found application in industrial environments [24]. 

Based on the reviewed studies, it can be concluded that the selection of the proposed three MADM methods introduces several innovations compared to existing approaches:

- Since research on base material selection for repair welding using MADM methods remains very limited in the relevant literature, it can be stated that this study introduces a new area of application for these methods;
- The BWM is applied for determining attribute weights, as it has proven to be very practical for engineering problems;
- The TOPSIS method is applied, which, although considered reliable, has not yet been used for the problem under consideration;
- A new application of the RADAR method is established, which, although recent, has proven reliable for solving other ranking problems;
- Methods based on different mathematical foundations can serve as a cross-check for the obtained results.

Ultimately, it can be stated that the proposed hybrid BWM-TOPSIS/RADAR approach provides a robust methodological basis for the selection of base materials for repair welding.

## 3. Proposed Hybrid MADM Approach

**Figure 1:**
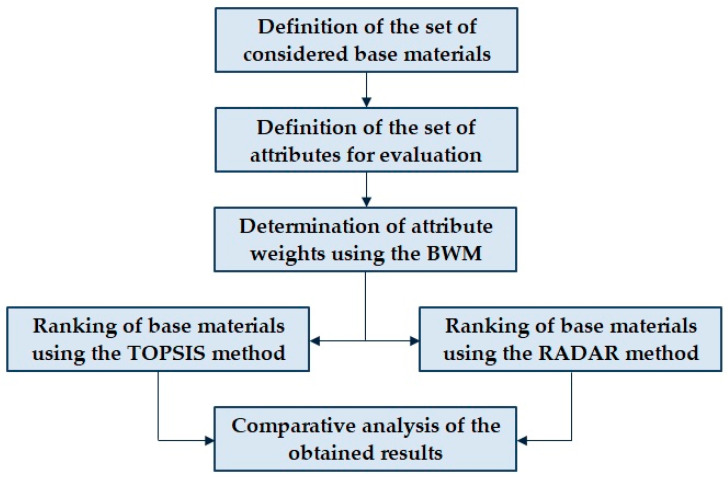
General overview of the proposed methodology.

Within this research, a two-stage model based on the application of three MADM methods was proposed. The BWM was used to determine the attribute weights, while the TOPSIS and RADAR methods were applied in parallel for ranking the base materials. In this way, the validity of the obtained results was examined. To provide a clearer understanding of the proposed model, a general overview of the methodology is presented in Figure 1.

In the following section of this chapter, the data collection process, the evaluation procedure conducted by experts, and the steps of the applied MADM methods are explained. 

### 3.1. Expert-Based Attribute Assessment Based on BWM

The problem addressed in this research is the evaluation and ranking of three available base materials for welding based on multiple attributes. The considered base materials are ABRADUR 58 (i=1), E DUR 600 (i=2), and CrWC 600 (i=3), and they can be formally represented by an index set i, i=1,…,I. Therefore, in this case, the base materials are considered as alternatives.

The attributes used for the evaluation and ranking of the base materials can be formally represented by an index set a, a=1,…,A, and they are wear track width (a=1), cost (a=2), mass loss (a=3), and hardness of welded layers (a=4). The first three attributes are of the cost type (where lower values are preferred), while the last attribute is of the benefit type (where higher values are preferred). A detailed description of these attributes is provided in the Section 4. Moreover, the data collection procedure and the measurements performed are explained in the Section 4.

A total of three experts, e, e=1,…,E, from the considered company participated in the process of selecting and evaluating the relative importance of the attributes. These experts possess adequate experience and knowledge in this field and were selected by the research team. They are the welding engineer (e=1), maintenance engineer (e=2), and operations manager (e=3).

The experts based their assessments on the standard procedure defined in the BWM [3]. First, they selected the best and worst attributes according to their own judgment. Then, they performed their evaluations using the standard scale from 1 to 9. The criterion weights obtained at the level of each expert were aggregated using the arithmetic mean operator.

The steps for applying the BWM are as follows [3]:

Step 1. Selection of the best and worst attributes at the level of each expert, e, e=1,…,E. The best attribute can be formally represented as $j_{B}^{e}$, while the worst attribute can be represented as $j_{W}^{e}$. 

Step 2. Evaluation of the best attribute in relation to all other attributes at the level of each expert, expressed on a scale from 1 to 9:

(1) $$j_{B}^{e}=(j_{B1}^{e},\ldots ,j_{Bj}^{e},\ldots ,j_{BJ}^{e})$$

Step 3. Evaluation of the worst attribute in relation to all other attributes at the level of each expert, expressed on a scale from 1 to 9:

(2) $$j_{W}^{e}={\left(j_{1W}^{e},\ldots ,j_{jW}^{e},\ldots ,j_{JW}^{e}\right)}^{T}$$

Step 4. The standard procedure defined in the BWM is applied to determine the Consistency Ratio (CR), and subsequently to calculate the attribute weights. In this way, the attribute weights at the level of each expert were calculated, $\left(\omega_{1}^{e},\ldots ,\omega_{j}^{e},\ldots ,\omega_{J}^{e}\right)$.

Step 5. The weights obtained from each expert are aggregated using the arithmetic mean operator to obtain the final, unified attribute weights, $\left(\omega_{1},\ldots ,\omega_{j},\ldots ,\omega_{J}\right)$:

(3) $$\omega_{a}=\frac{1}{E}\cdot \sum_{e=1}^{E}\omega_{a}^{e}$$

where a=1,…,A.

### 3.2. Selection of Base Materials Using the TOPSIS

The implementation steps were adopted from the conventional TOPSIS method [5]:

Step 1. Formation of the decision matrix. This matrix represents the value of each alternative with respect to each attribute:

(4) $${\left[x_{ij}\right]}_{IxJ}$$

Step 2. Formation of the normalized decision matrix. In this case, the normalization of values was carried out using the vector normalization procedure, regardless of the attribute type:

(5) $${\left[r_{ij}\right]}_{IxJ}$$

where

(6) $$r_{ij}=\frac{x_{ij}}{\sqrt{\sum_{i=1}^{I}x_{ij}^{2}}}$$

Step 3. Formation of the weighted normalized decision matrix:

(7) $${\left[z_{ij}\right]}_{IxJ}$$

where

(8) $$z_{ij}=r_{ij}\cdot \omega_{j}$$

Step 4. Determination of the Positive Ideal Solution (PIS), $z_{j}^{+}$. For benefit-type attributes, this represents the highest value of an alternative for the considered attribute. For cost-type attributes, it represents the lowest value of an alternative for the considered attribute.

For benefit-type attributes:

(9) $$z_{j}^{+}=\underset{i=1,\ldots ,I}{\max}z_{ij}$$

For cost-type attributes:

(10) $$z_{j}^{+}=\underset{i=1,\ldots ,I}{\min}z_{ij}$$

Step 5. Determination of the Negative Ideal Solution (NIS), $z_{j}^{-}$. For benefit-type attributes, this represents the lowest value of an alternative for the considered attribute. For cost-type attributes, it represents the highest value of an alternative for the considered attribute.

For benefit-type attributes:

(11) $$z_{j}^{-}=\underset{i=1,\ldots ,I}{\min}z_{ij}$$

For cost-type attributes:

(12) $$z_{j}^{-}=\underset{i=1,\ldots ,I}{\max}z_{ij}$$

Step 6. Determination of the distance of each alternative from PIS and NIS:

(13) $$d_{i}^{+}=\sqrt{\sum_{j=1}^{J}{\left(z_{ij}-z_{j}^{+}\right)}^{2}}$$

(14) $$d_{i}^{-}=\sqrt{\sum_{j=1}^{J}{\left(z_{ij}-z_{j}^{-}\right)}^{2}}$$

Step 7. Determination of the closeness coefficient of an alternative to the ideal solution:

(15) $$c_{i}=\frac{d_{i}^{-}}{d_{i}^{+}+d_{i}^{-}}$$

Step 8. Ranking of alternatives based on the closeness coefficient. Alternatives are arranged in a descending order, where the highest coefficient value indicates the best, and the lowest value indicates the worst alternative.

### 3.3. Selection of Base Materials Using the RADAR

The implementation steps were adopted from the conventional RADAR method [6]:

Step 1. Formation of the decision matrix. This matrix represents the value of each alternative with respect to each attribute:

(16) $${\left[x_{ij}\right]}_{IxJ}$$

Step 2. Formation of the maximum proportion matrix:

(17) $${\left[a_{ij}\right]}_{I\times J}$$

For benefit-type attributes:

(18) $$a_{ij}=\frac{\frac{\underset{i}{\max}x_{ij}}{x_{ij}}}{\left(\left(\frac{\underset{i}{\max}x_{ij}}{x_{ij}})+(\frac{x_{ij}}{\underset{i}{\min}x_{ij}}\right)\right)}$$

For cost-type attributes:

(19) $$\alpha_{ij}=\frac{\frac{x_{ij}}{\underset{i}{\min}x_{ij}}}{\left(\left(\frac{\underset{i}{\max}x_{ij}}{x_{ij}})+(\frac{x_{ij}}{\underset{i}{\min}x_{ij}}\right)\right)}$$

Step 3. Formation of the minimum proportion matrix. The elements of the minimum proportion matrix for benefit-type attributes are calculated in the same way as the elements of the maximum proportion matrix for cost-type attributes, and vice versa. The minimum proportion matrix can be formally represented as follows:

(20) $${\left[\beta_{ij}\right]}_{I\times J}$$

The following rule applies:

(21) $$a_{ij}+\beta_{ij}=1$$

Step 4. Formation of the empty range matrix:

(22) $${\left[E_{ij}\right]}_{I\times J}$$

where

(23) $$E_{ij}=|\alpha_{ij}-\beta_{ij}|$$

Step 5. Formation of the relative relationship matrix:

(24) $${\left[{RR}_{ij}\right]}_{I\times J}$$

where

(25) $${RR}_{ij}=\frac{\alpha_{ij}}{\beta_{ij}+E_{ij}}$$

Step 6. Formation of the weighted relative relationship matrix:

(26) $${\left[{WRR}_{ij}\right]}_{I\times J}$$

where

(27) $${WRR}_{ij}={RR}_{ij}\cdot \omega_{j}$$

Step 7. Determination of the aggregated ranking index value:

(28) $${RI}_{i}=\frac{\min\sum_{j=1}^{J}{WRR}_{i}}{\sum_{j=1}^{J}{WRR}_{i}}$$

Step 8. Ranking of alternatives based on the aggregated ranking index value. The coefficient ${RI}_{i}$ takes the value of 1 for the alternative ranked first. The remaining alternatives are ordered in descending order according to the value of this coefficient. The alternative with the lowest coefficient value is positioned last in the ranking.

## 4. Case Study

Within this section, a practical example of the application of the proposed methodology is presented [25]. For this purpose, the experiments were conducted within a company engaged in construction works. The company belongs to the group of small and medium-sized enterprises and is headquartered in Kragujevac, central Serbia.

**Figure 2:**
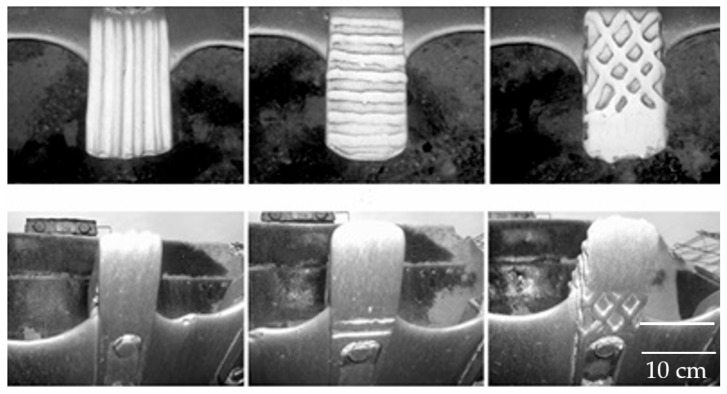
Example of some welded parts after service.

In this company, repair welding is used to restore working components and parts, most often of large-scale construction machinery. The goal is for the welded joints to perform identically to non-damaged components, maintaining their full functionality and applicability. Figure 2 shows the appearance of some welded parts after operational use.

In this specific case, an excavator bucket and the welded part of its teeth, which broke during operation, are presented. For the company, achieving savings is particularly important, as the service life of the bucket is extended, avoiding the need to invest in a new one.

### 4.1. Definition of the Set of Attributes for Base Material Selection

In collaboration with the management of the considered company, and primarily with the experts who participated in this research (welding engineer, maintenance engineer, and operations manager), the attributes based on which the base materials should be evaluated were established. The experts reached their decision by consensus and submitted it to the research team, after which they concluded that the most important criteria for selecting an adequate base material are as follows: (1) Wear track width [mm], (2) Cost [€/kg], (3) Mass loss [%], and (4) Hardness of welded layers [HV1].

Wear track width [mm] (a=1): The experimental investigation of wear resistance was conducted using a block-disk type tribometer (In-house developed technical solution at the Faculty of Engineering, University of Kragujevac, Kragujevac, Serbia), which allows simulation of real contact conditions, where a block-shaped sample is placed in contact with a rotating disk made of steel with known mechanical properties. The tribometer is designed to allow control of rotation speed, applied load, and contact duration. Tests were performed at a constant disk rotation speed and a constant load of several tens of newtons over a period of 60 min.

Before each test, the sample surfaces were polished to a defined roughness level and degreased with ethanol. At the contact point between the block and the disk, GLX-2 SEA 15W-40 lubricant was applied, simulating boundary lubrication conditions. During testing, the tribometer automatically recorded friction as a function of time, and after the test, the surfaces were visually and microscopically examined.

On the tested surfaces, the wear track appeared as an indentation (“crater”) resulting from micro-cutting and microplastic deformation of the material. The wear track width was measured using an optical microscope and a micrometer screw (UM-21 optical microscope, manufactured in the former USSR (Union of Soviet Socialist Republics)) at three points along the central line of the crater, and the mean value of these measurements was taken as the reference value. This measure represents a geometric criterion of wear resistance and allows comparison of materials independently of the friction coefficient. Comparison of wear track widths among different samples (weld deposits and base materials) showed that increasing material hardness led to a reduction in wear track width, confirming a direct correlation between microhardness and wear resistance.

Cost [€/kg] (a=2): The cost of the base material, expressed in euros per kilogram. The stated cost represents the average for the first and second quarters of 2025 in the Serbian market.

Mass loss [%] (a=3): The mass loss of the welded layers was determined by measuring the mass of the welded parts after welding, i.e., before use, and the mass after a certain period of operational use.

Hardness of welded layers [HV1] (a=4): The hardness of the welded layers and base material was determined using the Vickers method (HV1) on metallographic samples cut from the weld zone and cross-sections of characteristic blocks. Measurements were performed using a high-precision microhardness tester (Wolpert Testor HAT 1E hardness tester, manufactured by Wolpert, Sonneberg, Germany) under a load of 9.81 N. The indenter (a diamond pyramid with an angle of 136° between opposite faces) was pressed into the sample for 10 seconds, and the imprint dimensions were measured using an optical system at 400× magnification.

The used experimental parameters were obtained by procedure shown in details in our previous work [26], while microstructure and hardness values were measured in laboratory conditions for each specimen. The obtained results are than compared to the values given in certain standards for those materials as well as in producer’s recommendations.

### 4.2. Definition of the Set of Base Materials

Three available base materials on the market, selected for consideration by the company’s experts, were examined in this study. These materials were chosen because they are available on the market in the Republic of Serbia and meet the company’s basic quality requirements. The experts took into account factors such as the frequency of use of these base materials in practice, previous experience, and their technical characteristics.

The ABRADUR 58 electrode (i=1) is a special, heavily coated rutile electrode manufactured by SŽ–Elektrode Jesenice (Slovenske Železarne, Jesenice, Slovenia), designated as E 10-UM-60-GR according to the DIN EN 14700 standard [27]. It is intended for applying hard, highly wear-resistant layers on parts exposed to intense abrasive wear and moderate impact loads. The chemical composition of this electrode is characterized by a high carbon content (about 3.6%) and chromium content (about 32%), resulting in the formation of an extremely hard ledeburitic microstructure in the weld, with dominant chromium carbides of the Cr_7_C_3_ type. The hardness of the welded layer is approximately 58 HRC, corresponding to values of around 700 to 740 HV1. Due to this high hardness, the weld can only be machined by grinding. ABRADUR 58 is most commonly used for the repair and protection of working surfaces of excavator teeth, crushers, knives, plows, and similar parts subjected to purely abrasive wear.

The E DUR 600 electrode (i=2) is a basic, heavily coated electrode alloyed with chromium, used for hardfacing steels and steel castings exposed to combined abrasive and impact wear. This electrode, also from the SŽ–Elektrode Jesenice program, is designated E 6-UM-60 according to DIN EN 14700. Its chemical composition features a moderate carbon content (about 0.5%) and chromium content (about 7.5%), resulting in a martensitic microstructure with retained austenite and precipitated carbides. The hardness of the weld ranges from 56 to 60 HRC, providing a good combination of toughness and impact resistance. For this reason, E DUR 600 is most frequently used for the repair of jaw and rotary crushers, pneumatic and hydraulic tools, knives, plows, press tools, and parts of mining and construction machinery.

The third tested base material, CrWC 600 (i=3), is a basic, heavily coated electrode with high chromium and tungsten alloying. According to DIN EN 14700, it is designated E 10-UM-60-C, and its chemical composition includes approximately 4% carbon, 26% chromium, and 4% tungsten. These alloys enable the formation of a ledeburitic microstructure with a high proportion of primary and secondary carbides Cr_7_C_3_ and WC, providing exceptional resistance to purely abrasive wear. The hardness of the weld ranges from 60 to 62 HRC, or approximately 660 HV1. The weld is extremely hard and abrasion-resistant, yet brittle and sensitive to impact loads, so it is applied only to parts dominated by abrasive rather than impact wear. In practice, CrWC 600 is used for knives, crushers, rollers, and milling tools working in contact with hard mineral materials.

### 4.3. Application of BWM for Determining Attribute Weights

During the assessment of the relative importance of the attributes, the experts provided their evaluations independently. Each expert, using a scale from 1 to 9 and following the guidelines of the conventional BWM, assessed the ratio of the best attribute to all others, as well as the ratio of the worst attribute to all others. The obtained evaluations are presented in Table 1.

By applying the standard procedure defined in the BWM, the attribute weights were calculated for each expert, along with the corresponding CR. The values are presented in Table 2.

Based on the consistency verification procedure, it was determined that in all three cases the pairwise consistency level is acceptable.

The aggregated values of the attribute weights were determined using the arithmetic mean operator. The final, unified attribute weight values are:


$$\begin{array}{cccc} \omega_{1}=0.30 & \omega_{2}=0.18 & \omega_{3}=0.40 & \omega_{4}=0.12 \end{array}$$

In this specific case, it was assumed that all experts have equal importance in the decision-making process.

### 4.4. Application of the TOPSIS Method for Evaluation and Ranking of Base Materials

During the application of both the TOPSIS and RADAR methods, the decision matrix is first established. This matrix contains the values of each alternative with respect to each considered attribute. Table 3 presents the decision matrix, which serves as the input for these two methods. 

Through Steps 2 and 3 of the proposed algorithm, the decision matrix values are normalized and weighted (Table 4).

After that, the PIS and NIS were determined according to Steps 4 and 5 of the proposed algorithm, followed by the calculation of the distances from these values (Step 6):


$$\begin{array}{cc} d_{1}^{+}=0.074 & d_{1}^{-}=0.091\\ d_{2}^{+}=0.016 & d_{2}^{-}=0.129\\ d_{3}^{+}=0.133 & d_{3}^{-}=0.006 \end{array}$$

By applying Step 7 and Step 8, the values of the closeness coefficient were determined, and the alternatives were ranked, as presented in Table 5.

As the final result of applying the TOPSIS method, E DUR 600 was identified as the best material in the considered case.

### 4.5. Application of the RADAR Method for Evaluation and Ranking of Base Materials

As previously mentioned, the first step in applying both the TOPSIS and RADAR methods is the same, namely the formation of the decision matrix, i.e., the definition of the input data. In Steps 2 and 3 of the proposed RADAR algorithm, the maximum proportion matrix (Table 6) and minimum proportion matrix (Table 7) are formed.

Example of calculating the first element of matrices $a_{11}$ and $\beta_{11}$ (for cost type attribute):

$$\begin{array}{cc} a_{11}=\frac{\frac{0.445}{0.445}}{\left(\left(\frac{0.675}{0.445}\right)+\left(\frac{0.445}{0.445}\right)\right)}=\frac{1}{1.517+1}=0.397 & \beta_{11}=\frac{\frac{0.675}{0.445}}{\left(\left(\frac{0.675}{0.445}\right)+\left(\frac{0.445}{0.445}\right)\right)\;}=\frac{1.517}{2.517}=0.603 \end{array}$$

The subsequent steps (4 to 6) in applying the RADAR method are well-known and consistent with the conventional RADAR approach. Table 8 presents only the weighted relative relationship matrix.

Example of calculating Empty range matrix (Step 4) for the first element of the matrix:

$$E_{11}=\left|\alpha_{11}-\beta_{11}\right|=\left|0.397-0.603\right|=0.206$$

Example of calculating the first element of the relative relationship matrix (Step 5):

$${RR}_{11}=\frac{0.397}{0.603+0.206}=0.492$$

Example of calculating the first element of the weighted relative relationship matrix (Step 6):

$${WRR}_{11}=0.492\cdot 0.30=0.148$$

By applying Step 7 and Step 8, the values of the aggregated ranking index were determined, and the alternatives were ranked, as presented in Table 9.

Example of calculating the first element of the aggregated ranking index value (Step 7):

$${RI}_{1}=\frac{0.556}{0.736}=0.755$$

As with the TOPSIS method, the final result of applying the RADAR method identified E DUR 600 as the best material in the considered case.

The obtained rankings of the alternatives are identical when applying both methods. Even when comparing the coefficients, i.e., the closeness coefficients (TOPSIS) and the aggregated ranking index (RADAR), the results are highly consistent. In the considered case, the Pearson correlation coefficient of the examined coefficients is 0.98, indicating a very high degree of agreement. This fact indicates that the obtained results are sufficiently reliable, as they have been validated using two MADM methods with different mathematical foundations.

### 4.6. Sensitivity Analysis

For the purpose of validating the proposed model, a sensitivity analysis of the obtained alternative rankings was conducted by varying the attribute weights. These variations were performed by replacing the weight of one selected attribute with the weight of the most important attribute (j = 3), while the weights of the remaining two attributes were kept unchanged. The modified sets of attribute weights used in the analysis are presented in Table 10.

In the previous table, it can be seen that in Scenario 1, attribute j=1 had its weight replaced with that of the most important attribute j=3. In Scenario 2, j=2 had its value replaced with that of the most important attribute j=3, and in Scenario 3, j=4 had its value replaced with j=3. In each scenario, the weights of the remaining two attributes remained unchanged. In Table 11 and Table 12, the ranking of alternatives is presented for the baseline and the other three scenarios for TOPSIS and RADAR, respectively.

**Figure 3:**
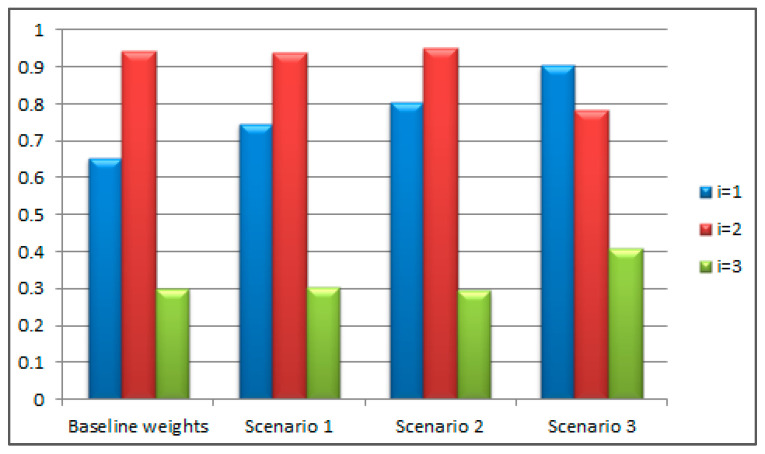
Average ranking coefficients per scenario using TOPSIS and RADAR.

Based on the results presented in Table 11 and Table 12, it can be concluded that the ranking of alternatives remains stable in two out of the three new scenarios. A change in ranking occurs only in Scenario 3, where the weights of the most important and least important attributes are directly swapped. However, in this case, some changes are to be expected. The lowest-ranked alternative remains in the same position, while the first and second alternatives switch places. The fact that Scenarios 1 and 2 maintain the alternatives in the same ranking positions indicates the robustness of the model against minor changes in attribute weights. Figure 3 shows the average value of the ranking coefficients obtained using the TOPSIS and RADAR methods for each considered scenario separately.

In Figure 3, it can be clearly observed that alternative a=1 gradually increases its average ranking coefficient as the weight of attribute j=3 gradually decreases (from 0.4 to 0.3, 0.18, and finally 0.12). This is because alternative a=2 performs significantly better than a=1 with respect to this attribute, while a=1 is better in terms of attributes j=1 and j=4. In fact, alternative a=1 only becomes first in the ranking when attributes j=2 and j=3, for which it performs worse than a=2, become the least important criteria. Therefore, the ranking result is completely logical and expected.

The Pearson correlation coefficient between the alternative rankings when comparing each TOPSIS scenario with each RADAR scenario is 1, i.e., there is no difference in the rankings. When all ranking coefficients are compared together, a very high Pearson correlation coefficient of 0.975 is obtained. This indicates a strong linear relationship between the ranking coefficients of the two methods, further demonstrating the robustness and reliability of the proposed model. 

## 5. Conclusions

In this study, a hybrid MADM approach was proposed based on two two-stage decision-making models. The first model consists of two phases, application of the BWM and TOPSIS method, while the second model involves the application of the BWM and RADAR method. The problem addressed in this paper is the selection of the optimal base material for repair welding. The BWM was used as a reliable basis for determining the attribute weights, as well as a method that is easily understood and straightforwardly applied by industry experts.

For the ranking and selection of the base material for repair welding, the TOPSIS and RADAR methods were employed, taking into account the attribute weights determined through the BWM, based on evaluations provided by experts from the examined company operating in the construction sector. A total of three materials with very similar characteristics were considered: ABRADUR 58, E DUR 600, and CrWC 600. The materials were evaluated according to the most important attributes for the company, namely wear track width, cost, mass loss, and hardness of welded layers.

Based on the conducted analyses, it can be concluded that the E DUR 600 electrode represents the best choice when technical, economic, and ecological criteria are considered. Although it is not the hardest among the tested electrodes, its microstructure provides an optimal balance between hardness and toughness, ensuring high resistance to combined abrasive and impact wear. Under operational conditions, components welded with this electrode exhibited the lowest mass loss (approximately 14%) and the most uniform wear, directly extending component service life and reducing maintenance frequency. In addition, E DUR 600 is also the most cost-effective electrode (≈7.49 €/kg), achieving the best price-to-durability ratio in relation to performance. Lower material consumption and longer service life also result in reduced waste and energy use, further supporting sustainable development principles.

Given that the results obtained through the BWM-TOPSIS and BWM-RADAR approaches are completely consistent, it can be concluded that this hybrid approach represents a highly reliable tool for solving this problem, as well as other engineering problems where it is necessary to balance multiple conflicting attributes or characteristics.

Although the proposed methodology offers several contributions and advantages, certain limitations should also be highlighted. The main contributions can be summarized as follows: (1) a high level of result reliability due to the consistency of rankings obtained by applying two mathematically different methods; (2) simple practical application, as the methodology does not require complex calculations; (3) flexibility of the model, which allows its application to other engineering problems; and (4) reliance on expert evaluations and experimental testing.

The main limitations of the proposed methodology are that (1) the accuracy of the model depends on expert assessments and the specific objectives of the company under consideration; (2) the existing assessments of attribute weights are valid only for the considered company; (3) a relatively small number of attributes was considered in the study; and (4) including other important characteristics (e.g., toughness/impact resistance, susceptibility to cracking, and dilution or compatibility with the base material) in the analysis may influence the final ranking of the alternatives.

Future research directions may include (1) extending the model by analyzing a greater number of alternatives and attributes; (2) introducing qualitative attributes, and consequently incorporating fuzzy logic into the problem; (3) application of continuous multi-criteria and optimization methods for solving the considered problem [28,29]; and (4) developing appropriate software based on the proposed model, which would enable experts to use it more easily and efficiently.

## Figures and Tables

**Table 1:** BWM-based expert assessments of attribute ratios.

Experts	Best-to-Others	Worst-to-Others
e=1	$j_{B}^{1}=\left(2,\;2,\;1,\;8\right)$	$j_{W}^{1}={\left(7,\;3,\;5,\;1\right)}^{T}$
e=2	$j_{B}^{2}=\left(2,\;2,\;1,\;5\right)$	$j_{W}^{2}={\left(3,\;2,\;5,\;1\right)}^{T}$
e=3	$j_{B}^{3}=\left(1,\;3,\;2,\;2\right)$	$j_{W}^{3}={\left(3,\;1,\;2,\;2\right)}^{T}$

**Table 2:** Calculated attribute weights and CR for each expert.

	e=1	e=2	e=3
Attribute weights	$\omega_{1}=0.27$	$\omega_{1}=0.21$	$\omega_{1}=0.42$
	$\omega_{2}=0.27$	$\omega_{2}=0.16$	$\omega_{2}=0.13$
	$\omega_{3}=0.41$	$\omega_{3}=0.55$	$\omega_{3}=0.23$
	$\omega_{4}=0.06$	$\omega_{4}=0.08$	$\omega_{4}=0.23$
CR	0.11	0.05	0.17

**Table 3:** Decision matrix for evaluated base materials.

	j=1	j=2	j=3	j=4
i=1	0.445	8.32	20	740
i=2	0.467	7.49	14	600
i=3	0.675	12.4	22	660

**Table 4:** Weighted normalized decision matrix.

	j=1	j=2	j=3	j=4
i=1	0.143	0.090	0.243	0.077
i=2	0.150	0.081	0.170	0.062
i=3	0.217	0.134	0.268	0.068

**Table 5:** Closeness coefficients and ranking of alternatives.

Alternative	Closeness Coefficient	Rank
i=1	0.552	2
i=2	0.889	1
i=3	0.044	3

**Table 6:** The maximum proportion matrix.

	j=1	j=2	j=3	j=4
i=1	0.397	0.427	0.565	0.448
i=2	0.421	0.377	0.389	0.552
i=3	0.603	0.623	0.611	0.505

**Table 7:** The minimum proportion matrix.

	j=1	j=2	j=3	j=4
i=1	0.603	0.573	0.435	0.552
i=2	0.579	0.623	0.611	0.448
i=3	0.397	0.377	0.389	0.495

**Table 8:** The weighted relative relationship matrix.

	j=1	j=2	j=3	j=4	Σ
i=1	0.148	0.107	0.400	0.082	0.736
i=2	0.171	0.078	0.187	0.120	0.556
i=3	0.300	0.180	0.400	0.120	1.000

**Table 9:** Aggregated ranking index and ranking of alternatives.

Alternative	Aggregated Ranking Index	Rank
i=1	0.755	2
i=2	1.000	1
i=3	0.556	3

**Table 10:** Sets of modified attribute weights used for sensitivity analysis.

	$\omega_{1}$	$\omega_{2}$	$\omega_{3}$	$\omega_{4}$
Baseline weights	0.30	0.18	0.40	0.12
Scenario 1	0.40	0.18	0.30	0.12
Scenario 2	0.30	0.40	0.18	0.12
Scenario 3	0.30	0.18	0.12	0.40

**Table 11:** Rankings of alternatives under different attribute weight scenarios—TOPSIS.

	Baseline Weights	Scenario 1	Scenario 2	Scenario 3
i=1	2 (0.552)	2 (0.665)	2 (0.763)	1 (0.807)
i=2	1 (0.889)	1 (0.880)	1 (0.898)	2 (0.649)
i=3	3 (0.044)	3 (0.044)	3 (0.041)	3 (0.172)

**Table 12:** Rankings of alternatives under different attribute weight scenarios—RADAR.

	Baseline Weights	Scenario 1	Scenario 2	Scenario 3
i=1	2 (0.755)	2 (0.826)	2 (0.847)	1 (1.000)
i=2	1 (1.000)	1 (1.000)	1 (1.000)	2 (0.918)
i=3	3 (0.556)	3 (0.566)	3 (0.548)	3 (0.647)

## Data Availability

The original contributions presented in this study are included in the article. Further inquiries can be directed to the corresponding author.

## Abbreviations

The following abbreviations are used in this manuscript:

AHP	Analytic Hierarchy Process
BWM	Best–Worst Method
CR	Consistency Ratio
MADM	Multi-Attribute Decision-Making
NIS	Negative Ideal Solution
PIS	Positive Ideal Solution
RADAR	RAnking based on the Distances And Range
SAW	Simple Additive Weighting
TOPSIS	Technique for Order of Preference by Similarity to Ideal Solution
